# Active Collision Avoidance for Human-Robot Interaction With UKF, Expert System, and Artificial Potential Field Method

**DOI:** 10.3389/frobt.2018.00125

**Published:** 2018-11-06

**Authors:** Guanglong Du, Shuaiying Long, Fang Li, Xin Huang

**Affiliations:** ^1^School of Computer Science and Engineering, South China University of Technology, Guangzhou, China; ^2^Guangzhou Start to Sail Industrial Robot Co., Ltd, Guangzhou, China

**Keywords:** active collision avoidance, artificial potential field method, unscented kalman filter (UKF), human-robot security, human-robot coexistence

## Abstract

With the development of Industry 4.0, the cooperation between robots and people is increasing. Therefore, man—machine security is the first problem that must be solved. In this paper, we proposed a novel methodology of active collision avoidance to safeguard the human who enters the robot's workspace. In the conventional approaches of obstacle avoidance, it is not easy for robots and humans to work safely in the common unstructured environment due to the lack of the intelligence. In this system, one Kinect is employed to monitor the workspace of the robot and detect anyone who enters the workspace of the robot. Once someone enters the working space, the human will be detected, and the skeleton of the human can be calculated in real time by the Kinect. The measurement errors increase over time, owing to the tracking error and the noise of the device. Therefore we use an Unscented Kalman Filter (UKF) to estimate the positions of the skeleton points. We employ an expert system to estimate the behavior of the human. Then let the robot avoid the human by taking different measures, such as stopping, bypassing the human or getting away. Finally, when the robot needs to execute bypassing the human in real time, to achieve this, we adopt a method called artificial potential field method to generate a new path for the robot. By using this active collision avoidance, the system can achieve the purpose that the robot is unable to touch on the human. This proposed system highlights the advantage that during the process, it can first detect the human, then analyze the motion of the human and finally safeguard the human. We experimentally tested the active collision avoidance system in real-world applications. The results of the test indicate that it can effectively ensure human security.

## Introduction

With the development of Industry 4.0, robots tend to be intelligent and cooperative in the future. On the one hand, robots can interact naturally with humans; On the other hand, robots can co-operate with people to produce in a common area. Based on this, the concept of human-computer collaboration emerged as a result, and was gradually sought after by industry, academia, and research institutions. In recent years, human-robot collaborative robots have also continued to mature and are used in some production workshops, such as UR5 for mass production lines. The human-machine collaboration feature will promote the wider use of robots and promote robots to play an indispensable partner role in human life (Yu et al., 2015; Li et al., 2017). However, according to the “Three Laws of Robotics,” the robot must not harm human beings or sit and watch human beings be harmed. It can be seen how important the role of robot security in the industrial development process is. As we all know, in order to ensure that the robot is safe enough, since the industrial robot was born more than half a century ago, most of the industrial robots have been placed in a static state, which is to determinate “isolated” environment, and accomplish a single repetitive task (Lee et al., 2017). Space is often isolated from people by very strong fences. Of course, let alone higher level of “into human life.” In recent years, with the deepening of the trend of Industry 4.0, the manufacturing industry has begun to develop the trend of customization, individuation and flexibility, which poses a severe challenge to the fixed production mode of traditional robots. This situation has led to inevitable close contact between robots and humans, and the traditional industrial robot production methods can't meet the needs of the company's production safety. The security considerations of collaboration between robots and humans have become the top priority for the future development of human-machine collaboration. The fact is that increasing the safety of robots often means compromises in performance. This requires designers to seek a balance between the two to ensure a win-win situation between safety and performance. Currently, collaborative robots have the ability to sense the environment and change their behavior according to the changes in the environment. For example, when a robot arm collides with a human arm, a human-robot cooperative robot can perceive the existence of a human arm according to a force sensor and stop and move away in time and do some other actions that protect human safety. Therefore, this function limits the performance of collaborative robots, such as speed, load, and so on. In addition, with the development of Industry 4.0, due to the large amount of traditional robots, it is obviously unrealistic to replace all existing traditional robots in a short period of time. Therefore, this paper proposes an effective method to predict the possibility of collision between robots and humans, and timely feedback to ensure human safety. Robots can successfully complete tasks in the well-known circumstances, such as factory working space. However, when a robot performs work in the dynamic, unstructured environments or even the environments with human, it has been a more challenging issue that how the robot works safely and efficiently (Nozaki et al., 2013). For the future robotics applications where humans and robots collaborate in carrying out tasks together (Karami et al., 2010), achieving safe and efficient human-robot interaction is indispensable. To meet the requirement of safety, it is necessary for robots to be able to find another path in the complex environment. In order that humans and robots coexist and collaborate in the complex environment, it is a big challenge at all times to provide human with safety protection. There are a few researches (Sadrfaridpour and Wang, 2017) which have been done to develop robot systems, including interaction and cooperation with humans in daily life. It is indicated that robots could be very useful to accomplish various tasks with humans jointly in these researches. Hence, it is always a big challenge to find an approach to protect human operators in human-robot collaborative systems, which includes two parts: detecting possible collisions in real time and human avoidance during runtime.

There are three parts (Flacco et al., 2012) in the active collision avoidance in real time: (1) Environment perception; (2) Collision avoidance algorithm; (3) Robot control. Collision avoidance is considered as one of the core technologies in robot research, which draws the widespread attention of the scholars. A lot of real-time capable planning concepts are based upon the potential field methods which are introduced in Zanchettin et al. (2016) and Minguez and Montano (2009). These methods use virtual attraction to represent targets and use repulsive field to represent obstacles. As a result, the robot will automatically get close to the targets and move away from the obstacles.

As to robotic systems, real-time planning algorithms (Harada et al., 2004; Saffari and Mahjoob, 2009) are of great significance. Due to the planning algorithms, robots are able to avoid the obstacles in real environment. A planning algorithm, which can coordinate humans and robots adaptively in hybrid assembly systems, has been proposed in method (Takata and Hirano, 2011). And according to method (Ohashi et al., 2007), the arm force is an advantage to detect the obstacles and avoid the objects in the environment.

Several researches have been done on obstacle avoidance in dynamic environments (Dietrich et al., 2012; Wu and Ma, 2013). The researches mostly treated humans as moving obstacles. In this case, the only thing to consider is the collision avoidance of robots. However, there are some other researches choosing not to treat humans as moving obstacles. What is worth mentioning, the assumption of the most of these above works is that the condition of the environment is available. Most of the collision avoidance algorithms are based on the distances between the robots and the objects in real environment.

In man-machine collaboration systems, in order to achieve the safety protection, the approaches of avoiding collision which have been applied are as follows: A novel meshless deformation model of biological soft tissue is presented for interactive simulation applications (Zou et al., 2017). Magneto-rheological fluid based compliant actuation mechanism (Ahmed and Kalaykov, 2010a,b), by introducing into robotic joint. Therefore robot need to work in a magnetic field environment. Vision-based method (Krüger et al., 2005), based on the analysis of motion, color and texture. Inertial sensor-based method (Corrales et al., 2011), using a special suit to capture motion. However, it is not practical for the inertial sensor-based method to be applied in the realistic environment, for the reason that a special-purpose suit with built-in sensors is required in this method. Moreover, it can only capture the wearer's motion leaving the other objects in the environment omitted. And this may cause severe safety problem because humans could be injured by the surroundings. Currently, visual-based method is proved to be a more practical approach for collision avoidance. Besides, as the optical 3D sensors developed, some newly-launched depth sensors, such as Microsoft Kinect (Microsoft Corporation, 2015), make developing a powerful sensor system with minimum effort available.

In recent years, the vision-based method has focused on the efficiency of collision detection. Virtual 3D models of robots and real camera images of operators are used for monitoring and collision detection, the control system can alert an operator, stop a robot, or modify the robot's trajectory away from an approaching operator by zero robot programming (Wang et al., 2013). A multi-camera system was adopted in Gecks and Henrich (2005) for detecting obstacles, whereas an emergency-stop based approach was employed in Ebert et al. (2005) to avoid a collision using an *ad-hoc* vision chip. In Tan and Arai (2011), a triple stereovision system was reported for tracking the movement of a sitting operator (upperbody only) by wearing color markers. However, due to uneven environmental lighting condition, mobile operators appearing in the monitored area may not show consistent colors. Instead of markers, a ToF (time-of-flight) camera was adopted in Schiavi et al. (2009) for collision detection, and an approach using multiple 3D depth images was presented in Fischer and Henrich (2009) for the same purpose. In method (Flacco et al., 2012) a new integrated approach was proposed to avoid collision by using one Kinect sensor and also raised depth space approach.

Although these methods have been notable performances in safety protection, our method has more necessary improvements.

1. Most researches treat humans as moving obstacles, so it is likely that humans are mistaken for an operation objects in the complex environment and may be injured, such as work pieces. While our method can detect the human, then analyze the motion of the human, and finally safeguard the human.
2. Most of the collision avoidance algorithms are based on the distances between the robots and the objects in real environment. While Our method can make an optimal reaction in the light of the speed of the human. When the human is approaching too fast, the robot keeps away from the human; when the human is approaching slowly, the object for the robot to avoid is the bounding sphere instead of the human; when the human is static and judged to impede the robot, a new path would be generated.
3. The avoidance time of our method is shorter because no continuous path calculation is required.

**Figure 1:**
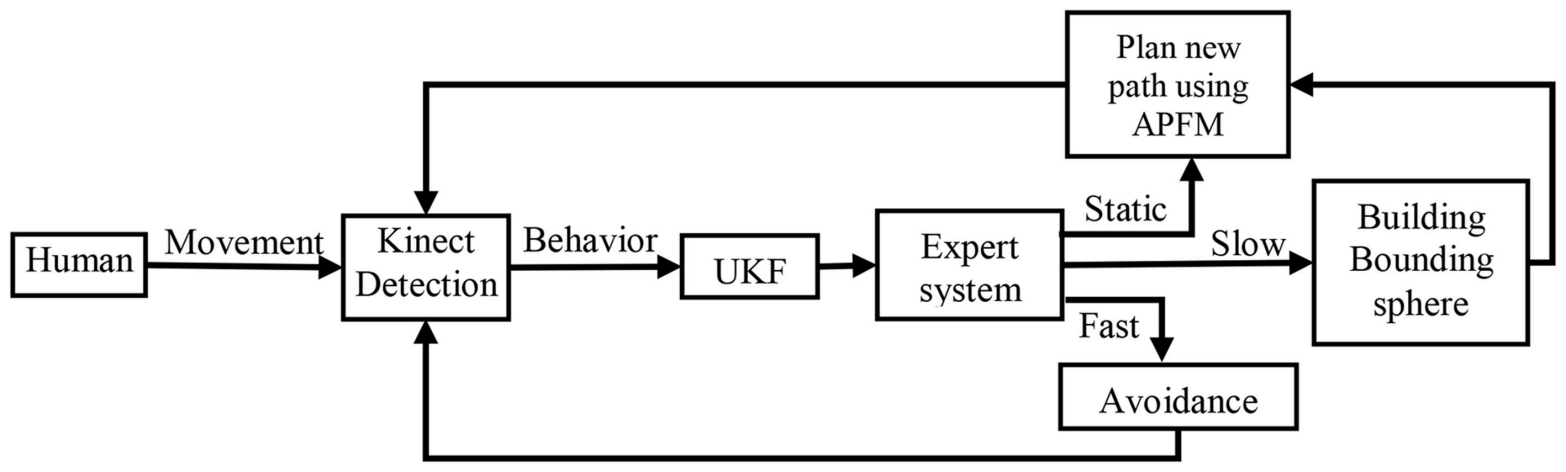
Process chart of the proposed method.

This paper proposed an active collision avoidance method (as shown in Figure 1). A Kinect is employed to detect the human in real time, since it can not only satisfy the demand of function but also be accessible. Most importantly, the human can be identified according to the characteristics of 3D data and the skeletons of the human can be calculated, even if the human is static, which greatly guarantees the safety of the human. UKF is applied to reduce the influence of time-varying and uncertainty of the signals of the position of the skeletons to complete human tracking. Because of the randomness of human movements, only using real-time path planning cannot realize the aim of active obstacle avoidance, so we use an expert system to analyze the behavior of the human. Depending on the status of human, we use artificial potential field method to plan a new path so that the robot can bypass the human in real time. When the human moves at a relatively low speed, the system calculates a bounding sphere and the robot bypasses the bonding sphere by using artificial potential field method to plan a new path. When the human is moving fast, the robot must avoid the human immediately. According to the current states of the human, an optimal reaction will be made, which saves much time. By taking these measures, this active collision avoidance method can not only achieve the goal that cutting down the avoidance time and the avoidance number at a large scale, improving the efficiency, but also solve the problem of the cost.

The remainder of paper is organized as follows. In section Human Identification, the human identification is described. The human tracking using unscented Kalman filter is detailed in section Human tracking using unscented kalman Filter and the collision avoidance is introduced in Collision Avoidance. Then we show the experiments and results in section Experiment. Discussions are presented in section Discussions before ending the paper with conclusions in section Conclusions.

## Human identification

There are two key design which takes driving the human movement tracking approach as a goal: computational efficiency and robustness. A single input depth image is segmented into a dense probabilistic body part labeling, with the parts defined to be spatially localized near skeletal joints of interest. We use an application program interface (API), which is embedded in the Kinect, to carry out human positioning and tracking. When human enters the workspace of the robot, the human can be detected according to the characteristics of 3D data and the skeletons of the human can be calculated by using the API.

**Figure 2:**
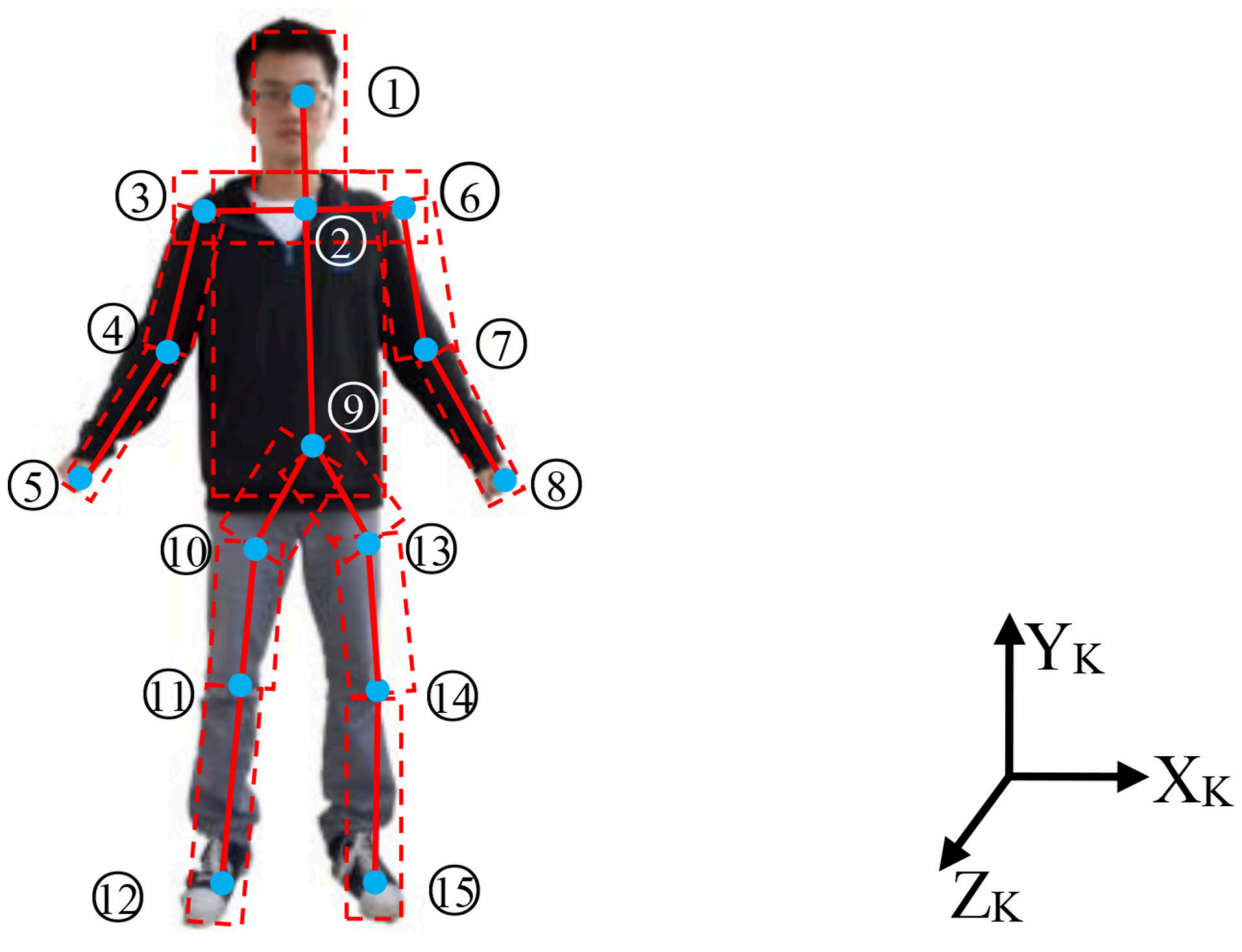
Skeleton joint points in the 3D space. (The person is Dolin and he consents for the Publication of the Manuscript.) 1, Head; 2, Shoulder center; 3, Right shoulder; 4, Right elbow; 5, Right wrist; 6, Left shoulder; 7, Left elbow; 8, Left wrist; 9, Hip center; 10, Right hip; 11, Right knee; 12, Right foot; 13, Left hip; 14, Left knee; 15, Left foot.

As we know, from Kinect we can derive the skeleton joint points. The 15 skeleton joints in the RGB image are shown in Figure 2. We number the 15 skeleton joint points from top to bottom and left to right. The coordinates of the 15 skeleton joint points are referred to as Kinect coordination. This method uses cylinder to construct the human body (Figure 2), since the human body has volume. Using the two adjacent points to build a cylinder is presented. According to the distance of the two adjacent points, we can determine the size (length and radius) of every cylinder, since the distance of the two adjacent points for every person is not the same. Furthermore, every cylinder of different part of human body is also not the same. The cylinder of the arm is smaller than the one of the trunks. By using the statistical data, we can calculate the length of the two corresponding adjacent points and the scaling relation between the sizes of a cylinder.

## Human tracking using unscented Kalman filter

Since the signals of the position of the skeletons are time-varying and they are ill-defined when occlusion is encountered, an adaptive filter is required.

### The unscented transform

The unscented transform determines the mean and variance of any random variable by using a set of sigma points (William and Aaron, 2009). Assume that *y* = *f*(*x*) is a non-linear transform function of variable *x*. The state vector *x* is an *n*-dimensional random variable with mean μ and covariance matrix *P*, χ_*i*_ represents the set of sigma points for the random variable *x*. Therefore, the statistics of the original random variable can be achieved by the 2n+1 sigma points and the corresponding weights *W*_*i*_, which is given as follows:

(1) $$\begin{array}{l} \chi_{0}=\bar{x}\;i=0\\ \chi_{i}=\bar{x}+{\left(\sqrt{(n+\lambda )P_{x}}\right)}_{i}\;i=0,...,n\\ \chi_{i}=\bar{x}-{\left(\sqrt{(n+\lambda )P_{x}}\right)}_{i-n}\;n+1,...,2n\\ W_{0}^{m}=\lambda /(n+\lambda )\\ W_{0}^{c}=\lambda /(n+\lambda )+(1-\alpha^{2}+\beta )\\ W_{i}^{m}=W_{i}^{c}=1/\{2(n+\lambda )\}\;i=1,...,2n \end{array}$$

where $\bar{x}$ is the mean of the sigma points, the number of the sigma points is n. λ = α^2^(*n* + *k*)−*n* is a composite scaling parameter, α controls the spread of the sigma points around $\bar{x}$. Making an adjustment for α can minimize the impact of higher-order terms. α is usually recommended to range from 0.0001 to 1. Although there is no specific range of *k*, it should be ensured that the matrix (*n* + λ)*P*_*x*_ is positive semi-definite matrix. In general, *k* = 0. As for a Gaussian distribution, *k* is chosen by *k* = 3−*n*. The parameter β is introduced to improve the accuracy of the covariance. As for a Gaussian distribution, β = 2 is optimal. ${\left(\sqrt{\left(n+\lambda \right)P_{x}}\right)}_{i}$ represents the column *i* of matrix $\left(\sqrt{\left(n+\lambda \right)P_{x}}\right)$.

Suppose that the estimated statistics of *y* can be determined by generating a set of sigma points. The estimated statistics of *y* are then related to the sample mean $\bar{y}$ and variance *P*_*y*_, which is defined

(2) $$\begin{array}{l} \begin{array}{c} y=f\left(\chi_{i}\right)\begin{array}{c} \;i=0,\ldots ,2n \end{array}\\ \bar{y}=\overset{2n}{\underset{i=0}{\sum }}W_{i}^{m}y_{i}\\ P_{y}=\overset{2n}{\underset{0}{\sum }}W_{i}^{c}\left(y_{i}-y\right){\left(y_{i}-y\right)}^{T} \end{array} \end{array}$$

Define (2n + 1)-dimensional vector χ as:

(3) $$\begin{array}{l} \chi =\left[\chi_{0},\chi_{1},\ldots ,\chi_{L},\chi_{L+1},\ldots ,\chi_{2n}\right] \end{array}$$

Since χ_*i*_(*i* = 0, 1, …2*n*) is a vector named σ vector, the specific form of σ is expressed

(4) $$\begin{array}{l} \left\{ \begin{array}{cc} \chi_{0}=\bar{x}\;\\ \chi_{i}=\bar{x}+\sqrt{\left(n+\lambda \right)}{\left\{chol\left(P_{x}\right)\right\}}_{i}^{T}\; & i=1,\ldots ,n\\ \chi_{i}=\bar{x}-\sqrt{\left(n+\lambda \right)}{\left\{chol\left(P_{x}\right)\right\}}_{i}^{T}\; & i=n+1,\ldots ,2n\\ \; \end{array}\right. \end{array}$$

where *chol*(*P*_*x*_) represents the Cholesky decomposition of *P*_*x*_, ${\left\{chol\left(P_{x}\right)\right\}}_{i}^{T}$ is the column *i* of the transpose of *chol*(*P*_*x*_).

The conversion result of each column vector of χ using the following nonlinear function is

(5) $$\begin{array}{l} y=f\left(\lambda_{i}\right)\begin{array}{c} i=0,1,\ldots ,2n \end{array} \end{array}$$

Then, the mean and variance of *y* = [*y*_0_,*y*_1_,…, *y*_2*n*_] can be expressed

(6) $$\begin{array}{l} \begin{array}{c} \bar{y}=\overset{2n}{\underset{i=0}{\sum }}\left(W_{i}^{\left(m\right)}y_{i}\right)\\ P_{y}=\overset{2n}{\underset{i=0}{\sum }}\left[W_{i}^{\left(c\right)}\left(y_{i}-\bar{y}\right){\left(y_{i}-\bar{y}\right)}^{T}\right] \end{array} \end{array}$$

### Unscented Kalman filter (UKF)

The unscented transform can be applied to Kalman filter (Hongzhong and Fujimoto, 2014) to estimate state. A general non-linear tracking system can be expressed as following

(7) $$\begin{array}{l} \begin{array}{c} x_{k+1}=F\left(x_{i},u_{k}\right)\\ y_{k}=H\left(x_{k},n_{k}\right) \end{array} \end{array}$$

where *x*_*i*_ is the state at time *k*, *F* is the state update function, and *H* is the observation function. *u*_*k*_ is the process noise and *n*_*k*_ is the observation noise.

The Kalman filter method which incorporates the unscented transform is achieved by the following processes.

1. Initialization state:
  (8) $$\begin{array}{l} \begin{array}{c} {\bar{x}}_{0}=E\left[x_{0}\right]\\ P_{0}=E\left[\left(x_{0}-{\bar{x}}_{0}\right){\left(x_{0}-{\bar{x}}_{0}\right)}^{T}\right] \end{array} \end{array}$$
2. Building expansion matrix:
  (9) $$\begin{array}{l} \chi_{k-1}=[{\hat{x}}_{k-1},{\hat{x}}_{k-1}+\sqrt{\left(n+\lambda \right)}{\left\{chol(P_{k-1})\right\}}_{i}^{T},\\ \;{\hat{x}}_{k-1}-\sqrt{\left(n+\lambda \right)}{\left\{chol(P_{k-1})\right\}}_{i}^{T}] \end{array}$$
  where the superscript indicates a value after applying the state transition function.
3. Time update:
  (10) $$\begin{array}{l} \;\chi_{k-1}=[{\hat{x}}_{k-1},{\hat{x}}_{k-1}+\sqrt{\left(n+\lambda \right)}{\left\{chol(P_{k-1})\right\}}_{i}^{T},\\ \;{\hat{x}}_{k-1}-\sqrt{\left(n+\lambda \right)}{\left\{chol(P_{k-1})\right\}}_{i}^{T}]\\ \chi_{k|k-1}=f(\chi_{k-1}) \end{array}$$
  χ_*k*|*k*−1_ = *f*(χ_*k*−1_) is the state transition function which is applied to the sigma points χ_*k*−1_, generating a new set of sigma points χ_*k*|*k*−1_. The estimated state ${\hat{x}}_{k|k-1}$ and the estimated covariance *P*_*k*|*k*−1_ are the weighted sample statistics of χ_*k*|*k*−1_ given by
  $$\begin{array}{l} {\hat{x}}_{k|k-1}=\overset{2n}{\underset{i=0}{\sum }}\left[W_{i}^{\left(m\right)}{\left(\chi_{k|k-1}\right)}_{i}\right] \end{array}$$
  (11) $$\begin{array}{l} P_{k|k-1}=\sum_{i\;=\;0}^{2n}\{W_{i}^{\left(c\right)}\left[{\left(\chi_{k|k-1}\right)}_{i}-{\hat{x}}_{k|k-1}\right]\\ \;{\left[{\left(\chi_{k|k-1}\right)}_{i}-{\hat{x}}_{k|k-1}\right]}^{T}\}+Q_{k} \end{array}$$
  where *Q*_*k*_ is system noise variance. Suppose the observation function *y*_*k*|*k*−1_ = *h*(χ_*k*|*k*−1_) generates a third set of sigma points, the estimated observation state ŷ_*k*|*k*−1_ and the estimated observation covariance $P_{k|k-1}^{o}$ are the weighted sample statistics of given by
  (12) $$\begin{array}{l} y_{k|k-1}=h(\chi_{k|k-1})\\ {\hat{y}}_{k|k-1}=\sum_{i\;=\;0}^{2n}\left[W_{i}^{\left(m\right)}{\left(y_{k|k-1}\right)}_{i}\right]\\ P_{k|k-1}^{o}=\sum_{i\;=\;0}^{2n}\{W_{i}^{\left(c\right)}\left[{\left(y_{k|k-1}\right)}_{i}-{\hat{y}}_{k|k-1}\right]\\ \;{\left[{\left(y_{k|k-1}\right)}_{i}-{\hat{y}}_{k|k-1}\right]}^{T}\}+R_{k} \end{array}$$
  where *R*_*k*_is observation noise variance.
4. Measurement update:
  (13) $$\begin{array}{l} P_{x_{k},y_{k}}=\overset{2n}{\underset{i=0}{\sum }}\left\{W_{i}^{\left(c\right)}\left[{\left(\chi_{k|k-1}\right)}_{i}-{\hat{x}}_{k|k-1}\right]{\left[{\left(y_{k|k-1}\right)}_{i}-{ŷ}_{k|k-1}\right]}^{T}\right\}\\ K_{k}=P_{x_{k},y_{k}}P_{{\bar{x}}_{k},{\bar{y}}_{k}}^{-1} \end{array}$$
  where *P*_*x*_*k*_, *y*_*k*__ is the sample cross correlation of χ_*k*|*k*−1_ and *y*_*k*|*k*−1_ *K*_*k*_ is the Kalman gain.
  The estimated state and covariance are as follows:
  (14) $$\begin{array}{l} \begin{array}{c} x_{k}={\hat{x}}_{k}+K_{k}\left(y_{k}-{\hat{y}}_{k}\right)\\ P_{k}=P_{k}^{-}-K_{k}P_{{\bar{x}}_{k},{\bar{y}}_{k}}K_{k}^{T} \end{array} \end{array}$$

### Skeleton points estimation using UKF

**Figure 3:**
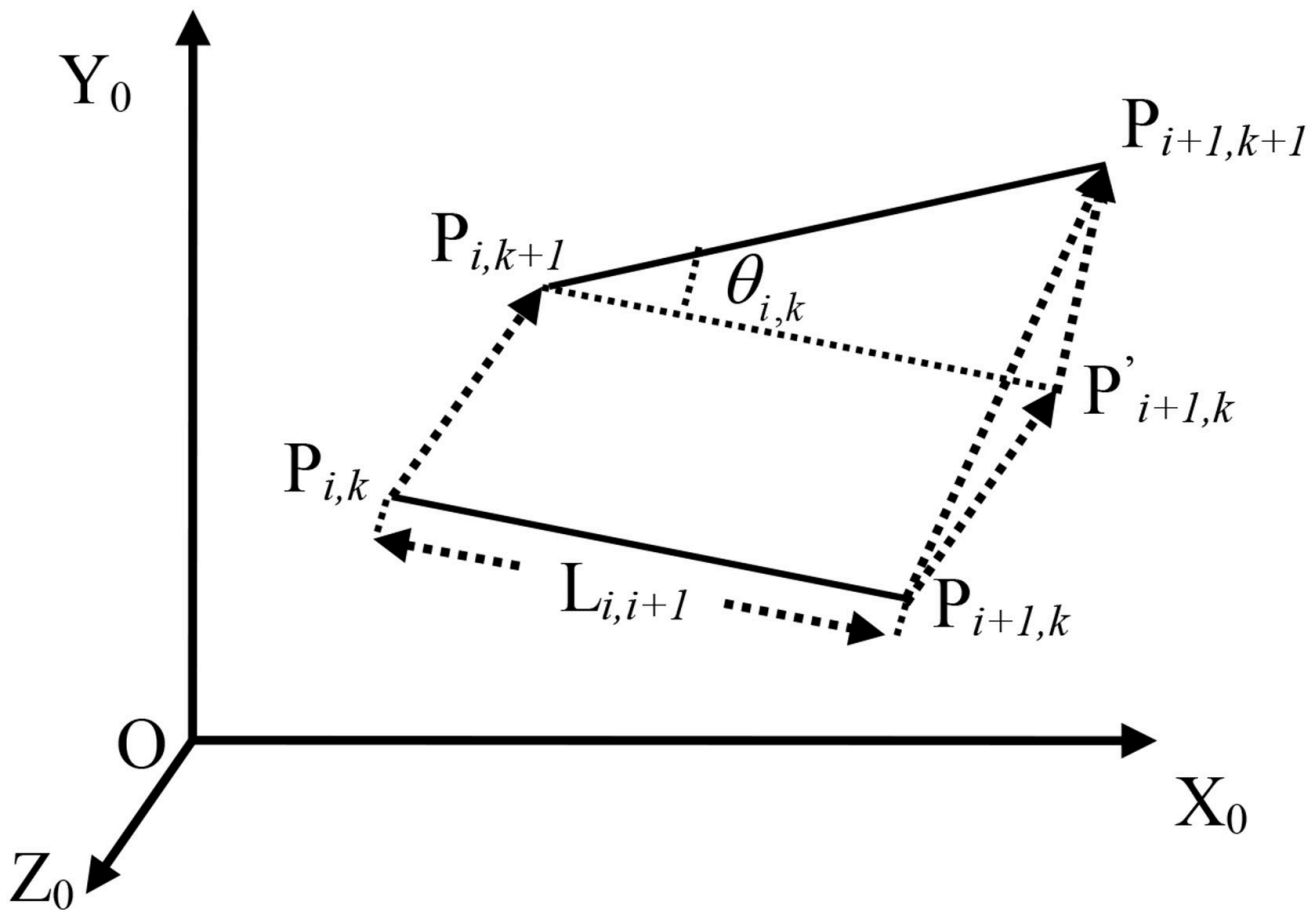
The status of points from *i* to *i*+*1*.

In the section II, there are fifteen skeleton points can be detected. In this section, we use UKF to estimate the skeleton points. From Figure 2 we can see that the skeleton points have been numbered from 1 to 15. Except the number 1, the other points have a father node (for example: the father note of point 3 is point 2; the father note of point 6 is point 2). Let *P*_*i,k*_, *P*_*i* + 1, *k*_ be the position of point *i*, *i* + 1 at time *k* with respect to coordinate of Kinect, *P*_*i,k*_ is the father note of *P*_*i* + 1, *k*_. *t* is the sampling interval. Figure 3 shows the position of *P*_*i*_,*P*_*i*+1_ in time *k* and *k* + 1. At time *k* + 1, the position of *P*_*i*+1_ is:

(15) $$\begin{array}{l} P_{i+1,k+1}=P_{i,k+1}▪T(\vec{P_{i,k+1}P_{i+1,k}^{\prime }})•R(\theta_{i,k})\\ \;=P_{i,k+1}▪T(\vec{P_{i,k}P_{i+1,k}})•R(\theta_{i,k}) \end{array}$$

where *T* is the translation matrix and *R* is the rotation matrix. If *P*_*i,k* + 1_ can be calculated at time *k*+1, then *P*_*i*+1,*k*+1_ can be computed. In fact, all the points except the first point have a father point. If the first point *P*_1,*k*+1_ can be estimated, then the other points can be calculated by Eq. So the state of the UKF can be defined as

(16) $$\begin{array}{l} x_{k}=\left[P_{1,k},v_{1,k},p_{2,k},\theta_{2,k},\ldots ,p_{i,k},\theta_{i,k},\ldots ,P_{15,k},\theta_{15,k}\right] \end{array}$$

where *v*_1,*k*_ = [*v*_*x*_, *v*_*y*_, *v*_*z*_] is the velocity of the first point *P*_1,*k*_, θ_*i,k*_ is the rotation angle of *P*_*i*+1,*k*+1_ relative to *P*_*i,k*+1_ with respect to coordinate *X*_0_*Y*_0_*Z*_0_.

Define the rotation ϕ_*i,k*_ about the *x*_0_ axis as the roll of *P*_*i*_ in time *k*, the rotation Γ_*i,k*_ about *y*_0_ axis as the pitch and the rotation ψ_*i,k*_ about *z*_0_ axis as the yaw, then θ_*i,k*_ = [ϕ_*i,k*_, Γ_*i,k*_, ψ_*i,k*_]. According to Euler's theorem (Diebel, 2006) of finite rotations, the conversion from Euler angles to quaternion is:

(17) [qi,k0qi,k1qi,k2qi,k3]=[cos(ϕi,k/2)cos(Γi,k/2)cos(ψi,k/2)+sin(ϕi,k/2)sin(Γi,k/2)sin(ψi,k/2)sin(ϕi,k/2)cos(Γi,k/2)cos(ψi,k/2)-cos(ϕi,k/2)sin(Γi,k/2)sin(ψi,k/2)cos(ϕi,k/2)sin(Γi,k/2)cos(ψi,k/2)+sin(ϕi,k/2)cos(Γi,k/2)sin(ψi,k/2)cos(ϕi,k/2)cos(Γi,k/2)sin(ψi,k/2)-sin(ϕi,k/2)sin(Γi,k/2)cos(ψi,k/2)]

And the four Euler parameters are constrained as:

(18) qi,k02+qi,k12+qi,k22+qi,k32=1

where qi,k0 is a scalar and (qi,k1,qi,k2,qi,k3) is a vector. So the direction cosine matrix *R*(θ_*i,k*_) from the father frame to the child frame is represented as:

(19) R(θi,k)=[qi,k02+qi,k12−qi,k22−qi,k322(qi,k1q2+qi,k0qi,k3)2(qi,k0qi,k2+qi,k1qi,k3)2(qi,k1,qi,k2+qi,k0qi,k3)qi,k02−qi,k12+qi,k22−qi,k322(qi,k2,qi,k3+qi,k0qi,k1)2(qi,k1,qi,k3−qi,k0qi,k2)2(qi,k0qi,k1+qi,k2qi,k3)qi,k02−qi,k12−qi,k22−qi,k32]

Subscript *i* represents the point number, but not the parent child relation. The parent child relation can be known through Figure 2. *P*_1,*k*+1_ can be calculated as

(20) $$\begin{array}{l} P_{1,k+1}=P_{1,k}+v_{1,k}▪t \end{array}$$

The state update function can be defined as Equation 15 and Equation 20. Since the position of the points with respect to coordinate *X*_0_*Y*_0_*Z*_0_ can be measured by Kinect, then the observation function can be set as

(21) $$\begin{array}{l} H=\left[1,0,1,0,\ldots ,1,0\right] \end{array}$$

## Collision avoidance

### Fast path planning using artificial potential field method

Artificial potential field method consists of virtual attractive and repulsive field, which are used to represent targets and 5 obstacles. For each moving obstacle, a dynamic constraint (Ren et al., 2005) of the form can be employed:

(22) $$\begin{array}{l} f_{j}\left(q\right)={\left|q-P_{j}\right|}^{2}-D_{obs}^{2}\geq 0 \end{array}$$

where *q* is the position of the robot, *P*_*j*_ is the position of the moving obstacle *j. D*_*obs*_ is the protective distance of each detected obstacle. When the obstacle is far away from robot, virtual repulsive field (22) is ignored by the planning algorithm. When the distance between the moving obstacle and the robot are less than the protective distance *D*_*obs*_, the constraints become activated and the robot will move along the feasible motion directions. If unfortunately, all the motion directions are blocked by the obstacles, the robot will stop until the human moves away.

### Active collision avoidance using expert system

Since there is randomness in the human movement, only using real-time path planning is not able to achieve the purpose of active obstacle avoidance. Therefore, we adopt an expert system into this collision avoidance system. An expert base is used in the expert system, which actually is a decision-making method (Ren et al., 2005; Won et al., 2010). There are expert knowledge and rules contained in the knowledge base.

The proposed approach uses the expert system in three cases depending on the behavior of the human in the workspace of the robot (*D*_*HR*_ represents the distance between the human and the robot, which is less than *D*_*HR*_min_ the dangerous distance):

Case 1: The human is approaching too fast. When the human approaches the manipulator at the speed *v*_*H*_ > *v*_*H*_*danger*_ m/s (in which *v*_*H*_*danger*_ is the dangerous speed), the new path which is planned by the system cannot guarantee the safety of the human in the next second time. The best decision is to make the robot keep away from the human, instead of planning a new path at once.

Case 2: The human is approaching slowly. When the human approaches the manipulator at the speed (0 < *v*_*H*_ ≤ *v*_*H*_*danger*_m/s), by using artificial potential field method, the system needs to predict the human motion trail and generate a new path to avoid the human. Since human behavior is uncertain, a bounding sphere containing the entire possible motion trail in a period will be calculated by the system. In this case, the object for the robot to avoid is the bounding sphere instead of the human. If the human accelerated (*v*_*H*_ > *v*_*H*_*danger*_ m/s) all of a sudden, the system should react with case 1.

Case 3: The human is static. At the beginning, the system makes a judgment whether the human will impede the movement of the robot or not. If the impediment exists, a new path should be generated by using artificial potential field method. Since the human is static, there is no need for the robot to avoid a bounding sphere; a shorter, more efficient path is planned by the system. If the human moved at the speed *v*_*H*_ > *v*_*H*_*danger*_ m/s all of a sudden, the system should react with case 1. While the speed (*v*_*H*_ ≤ *v*_*H*_*danger*_ m/s), the system should react with case 2.

## Experiment

### Environment of experiment

To verify the proposed active collision avoidance system, we carried out a series of experiments in real-world application. Our method was compared with method (Flacco et al., 2012) in term of time and efficiency. To finish the experiments, we executed the proposed system on an eight-core CPU. Among the eight cores, four cores were for the visualization and the control of the robot movement and the other four were for the calculation of complex position and planning path.

**Figure 4:**
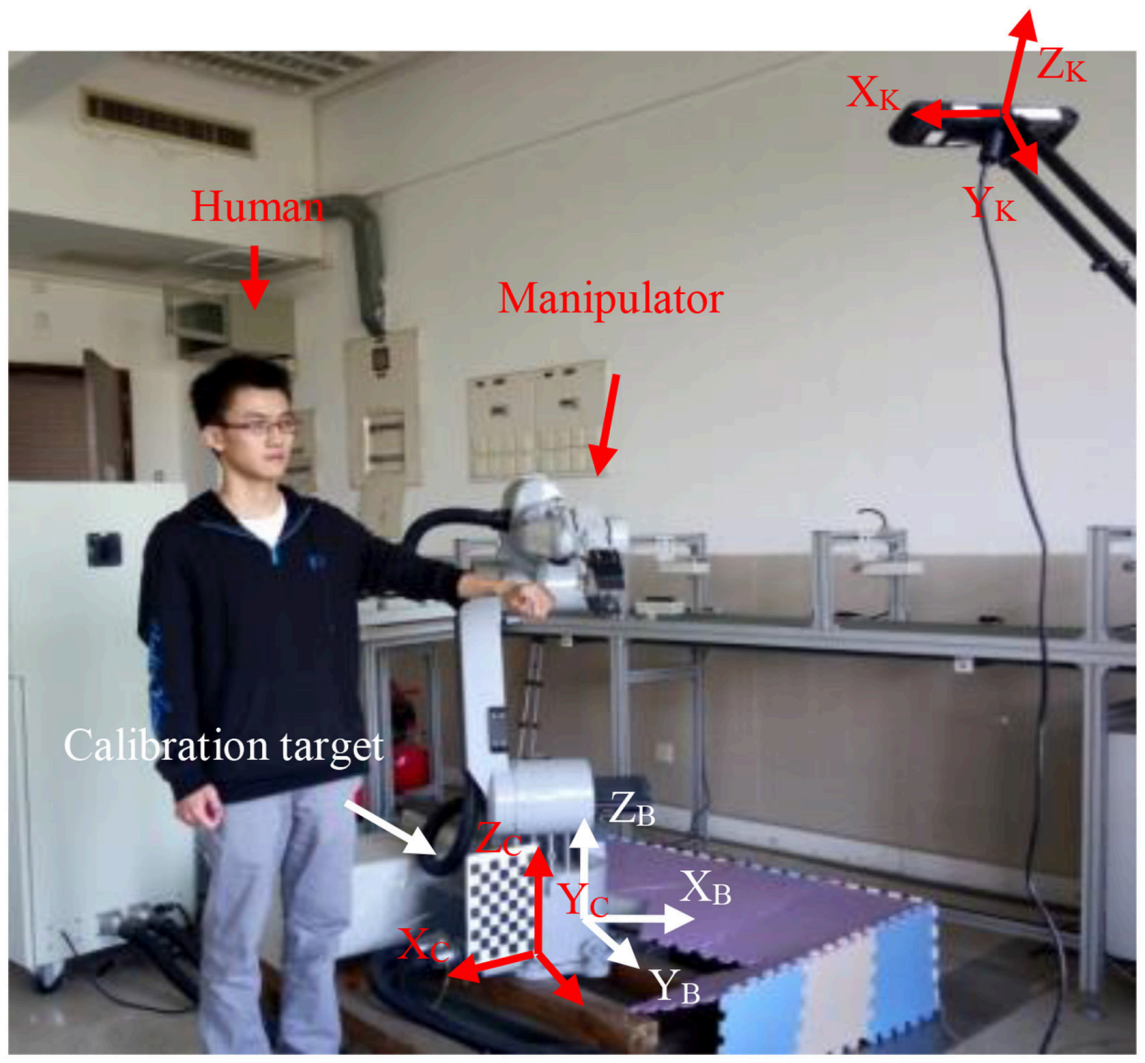
Experiment environment (the person is Dolin and he consents for the Publication of the Manuscript).

**Figure 5:**
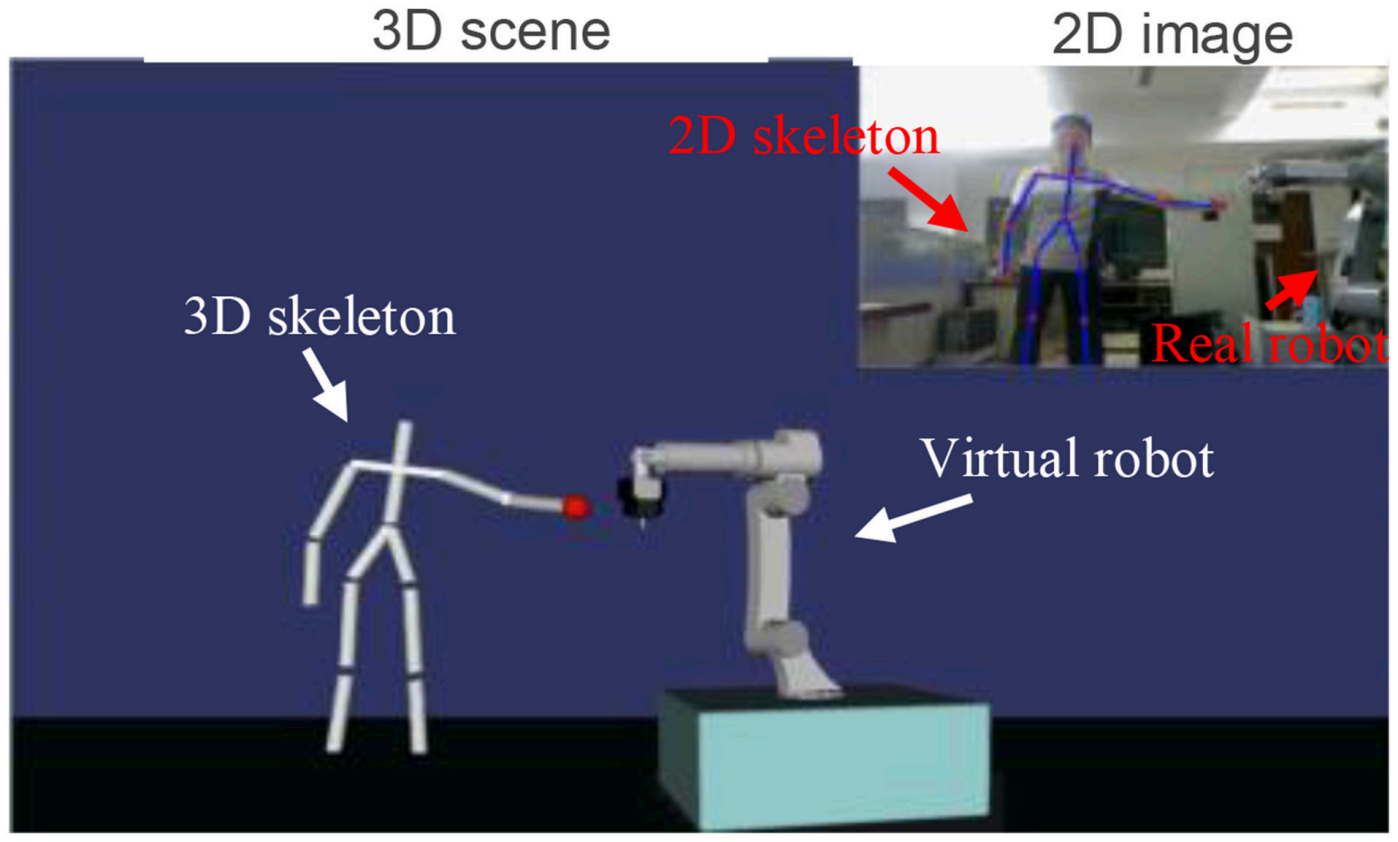
Experimental scene (the person is Jetty and he consents for the Publication of the Manuscript).

In the experiments, we used a GOOGOL GRB3016 robot with 6 DOF (degree of freedom). Table 1 shows the link parameters in D-H model of the robot. As to the robot control system, we adopted the reverse kinematics algorithm (Antonelli et al., 2003) to design a working path and controlled the robot to move along the path repetitively. The Brain Storm Optimization Algorithm also helps in designing a working path (Li et al., 2018; Song et al., 2018). Furthermore, we built an emulation scene including the robot manipulator model and the human model (**Figure 5**) in the robot control system. For the purpose of locating the 3D positions of the human and the robot, we designed a system to measure the positions with a Kinect and a calibration board. The Kinect was firmly fixed to a tripod, which is placed 1.6 meters away in vertical distance, 2.1 meters away in horizontal distance and 1.6 meters height with respect to the robot base and the calibration board was tightly attached to the robot near the robot base. The Kinect we used in experiments is Kinect1.0, which can detect the human with the built-in infrared camera. The resolution of the Kinect depth images was 320 × 240 pixels and the capture frequency is 30 Hz. The capture field of Kinect can include the working space of the robot. The distance for a human detection using Kinect is from 1.0 to 3.0 m. The angle of the Kinect should be adjusted appropriately before the experiments, to ensure that the capture of the human skeleton and the calibration board in real time could be successfully carried out during the experiments. The position relation of the Kinect and the robot base is determined by the calibration board. Human Assume that (*x*_*k*_, *y*_*k*_, *z*_*k*_), (*x*_*b*_, *y*_*b*_, *z*_*b*_) and (*x*_*c*_, *y*_*c*_, *z*_*c*_) are the frames of the Kinect, robot base and the calibration board respectively. We attached a calibration target to the robot base (Figure 4) rigidly. Method (Diebel, 2006) was used to calculated the relative location between the Kinect frame *X*_*K*_*Y*_*K*_*Z*_*K*_ and the calibration target frame *X*_*C*_*Y*_*C*_*Z*_*C*_. Then, we used a ruler to measure the relative location between the robot base frame *X*_*B*_*Y*_*B*_*Z*_*B*_ and the calibration target frame *X*_*C*_*Y*_*C*_*Z*_*C*_. In this case, with the help of a calibration target, we could determine the Kinect frame *X*_*K*_*Y*_*K*_*Z*_*K*_, with respect to the robot base frame *X*_*B*_*Y*_*B*_*Z*_*B*_. Before the experiments, we should measure and determine (Ping and Guang-long, 2011; Guanglong and Ping, 2013) the transformation from the robot base to the calibration board. In the experiments, by capturing the images of the calibration board with the Kinect, we could determine the position of the robot by detecting the corners of the calibration board. Moreover, we could obtain the positions of the human by the depth information from the Kinect.

**Table 1:** The link parameters in D-H model for the GOOGOL GRB3016 robot.

** 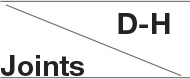 **	**a**	**α**	**d**	**θ**
1	150	−π/2	250	0
2	570	−π	0	−π/2
3	150	π/2	0	0
4	0	−π/2	650	0
5	0	−π/2	0	−π/2
6	0	0	−200	0

The goal of the experiments is to keep the robot from crashing into the human who entered the workspace of the robot. The parameters which are used for the experiment are: *v*_*H*_*danger*_ = 0.2 m/s, *v*_*R*_max_ = 2.0 m/s, *D*_*HR*_min_ = 0.2 m, where *v*_*R*_max_ is the maximum speed of the robot.

**Figure 6:**
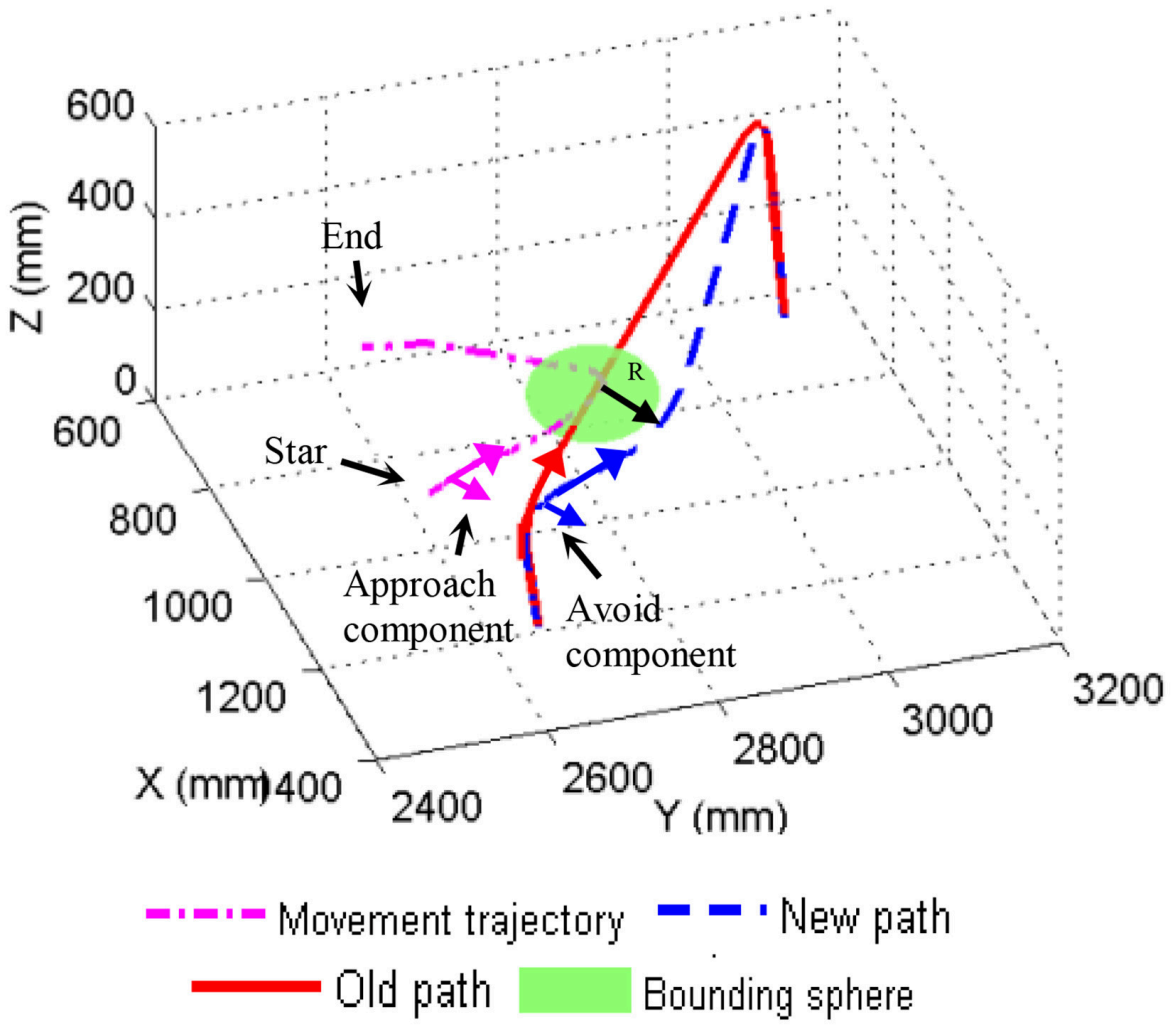
The 3D trajectory.

During the experiments, the robot moved along the designed path all the way (as shown in Old path of **Figure 6**) and the robot control system calculated the position of the robot EE with respect to the Kinect frame in real time. The robot control system calculated the position of the robot EE by substituting perθ into the D-H transformation matrix, and multiply by the six D-H transformation matrices. By the changed position of the end of the manipulator and the time it takes, the velocity of one can be obtained. When a person entered the workspace, the Kinect captured the skeleton of the person instantly. Therefore, according to the captured human skeleton, the robot control system was able to determine the human moving speed as well as the human position with respect to the Kinect frame. The moment human was found to approach robot, depending on the human moving speed, the system performed the collision avoidance. For the purpose of verifying the proposed method, we carry out the experiment with the human moving at the different speeds. The process of the experiment is showed as follows: Firstly, the human got close to the robot at a relative biggish speed which was more than 0.2 m/s. Afterwards, the human moved to the robot at the speed between 0 and 0.2 m/s. At the end, the human left the robot.

### Results of experiment

In the experiment, as we planned, a human attempted to interrupt the works of the robot. The experimental scene is shown in Figure 5. A 3D virtual scene was built, in order to monitor the environment around the robot. There was nothing but a virtual robot in the Initializing virtual scene. When a human was detected by the Kinect sensor, the 3D skeleton of the human would be calculated and then the 2D skeleton would be drawn on the 2D image and 3D skeleton in the virtual scene. In the virtual scene, the red ball was the closest point of the human with respect to the robot.

The 3D trajectory of a human and the robot is shown in Figure 6. The human tried to approach the robot and then leave the robot. The red line is the robot's old path. The dash-dot line is the human movement trajectory. The dashed line is the robot's new path. To start with, the human approached the robot fast and the system detected that the approaching speed of the human was more than *v*_*H*_*danger*_, then the robot made a judgment to avoid the human directly. Figure 6 shows that the avoidance component of the robot is equal to the approach component of the human. A bounding sphere was calculated by the system, in order to plan a new path for the robot, when the human slowed down.

**Figure 7:**
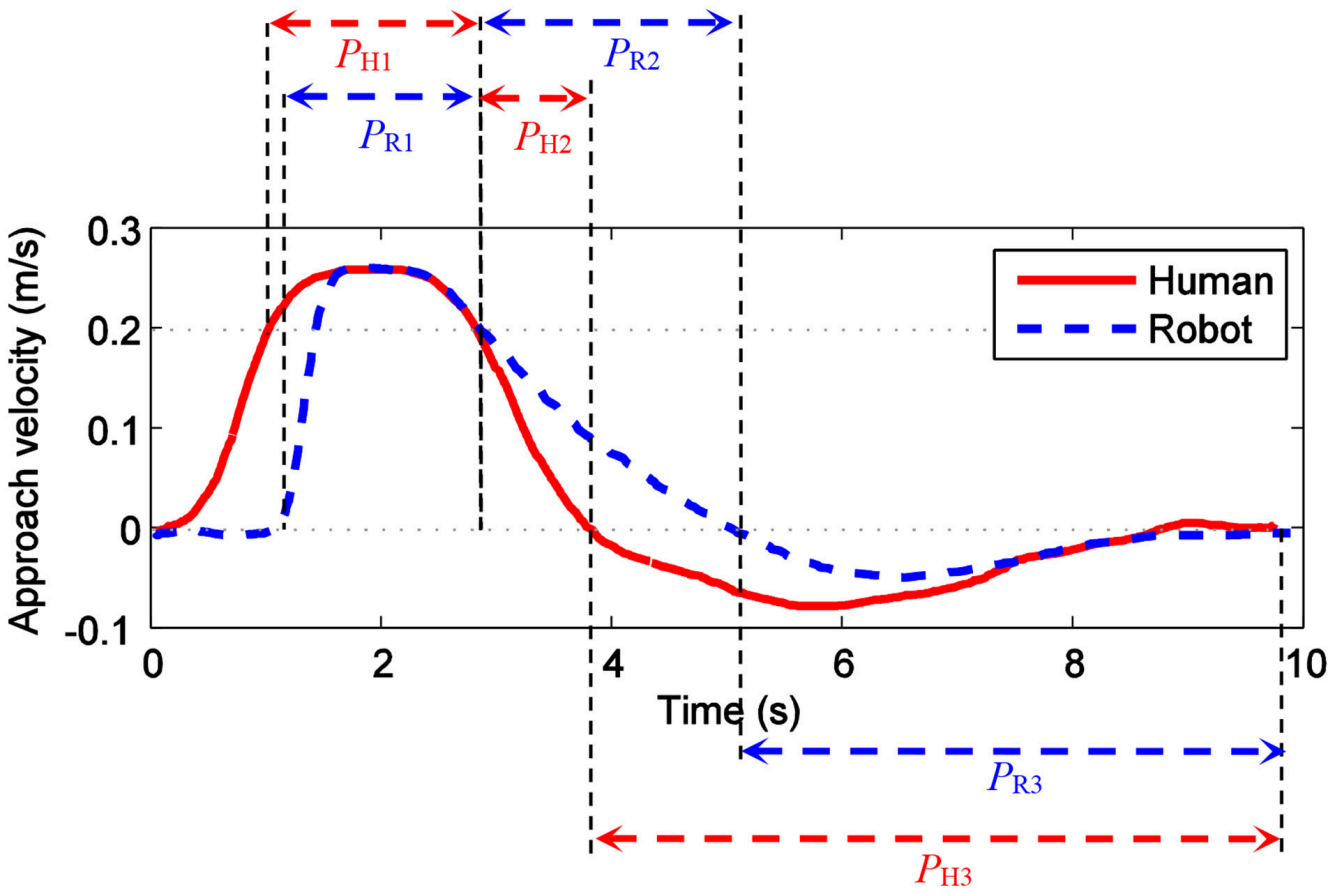
The velocity curve of the human and the robot in the direction of the connections of the human and the robot.

The velocity curve of the human and the robot in the direction of the connections of the human and the robot are shown in Figure 7. At the period between 0 s to 1.1th s, the human got close to the robot at an increasing speed. The avoidance component of the robot is 0, since the approach component is less than *v*_*H*_*danger*_. In the period *P*_H1_ between 1.1st s to 2.9th s, the approach component is more than *v*_*H*_*danger*_, and the robot accelerated in order to avoid the human. The avoid component and the approach component should be equal. The human slowed down after 2.9th s (*P*_H2_). After 3.9th s (*P*_H3_) the human left the robot. The avoid component keeps positive until 5.1st s (*P*_R2_), since the robot needed to avoid the human by bypassing the bounding sphere. Then the robot moved close to the old path (*P*_R3_) after the human left the robot.

After the experiment, our method was compared with the method (Flacco et al., 2012). In our method and method (Flacco et al., 2012), one Kinect sensor is used to monitor the workspace of robot for avoid collision. Method (Flacco et al., 2012) evaluates distances between the robot and possibly moving obstacles (including humans) based on the concept of depth space and use the distances to generate repulsive vectors that are used to control the robot while executing a generic motion task. While Our method can make an optimal reaction according to the current state of the human by expert system and artificial potential field method. When the human moved close to the robot, in our method, robot took avoidance measures directly, while in method (Flacco et al., 2012) a new path for the robot was constantly planned. In the period of the human slowing down, the way to avoid the human in our method was to avoid a bounding sphere, but in method (Flacco et al., 2012) the system had to plan a new path for the robot again and again since the human blocked the robot's path continuously. On one hand, Because the way of collect information in our method and method (Flacco et al., 2012) is the same, this ensures that the difference in experimental results is only caused by different methods. On the other hand, by comparison, we can know the impact of speed on collision avoidance.

Table 2 shows the comparative results between our method and method (Flacco et al., 2012). The avoidance time means the time during the robot's deviating from the old path to plan the new path until returning to the old one, and the avoidance numbers means the numbers of avoiding the human and planning the new path in our method and the numbers of planning the new path in method (Flacco et al., 2012) during the avoidance time. In method (Flacco et al., 2012), both the number of the avoidance and the avoidance time is increased. A new path for the robot was planned again and again owing to only considering the distance between the robot and the human in method (Flacco et al., 2012), more planning path needs and much calculation time needs. In method (Flacco et al., 2012), the summary number of planning path is 5 and the avoidance time is 12.7 s. By comparison, in our method, the system avoided the human twice and planned new path once, so the total count of avoidance is 3 and the avoidance time is 5.1 s. To avoid the human, the robot moved in a newly-planned path, the route increased compared with the old path. In method (Flacco et al., 2012), the increased route is 963 mm, while that of our method is 976 mm. When the human slowed down, a bounding sphere is calculated by our system so that the robot can move around the human fast, which results in shorter avoidance time. But in the method (Flacco et al., 2012), the system calculated a new path for the robot again and again so that the robot could move to avoid the human. The avoidance time of our method is less than half of method (Flacco et al., 2012), despite the shorter increased route of method (Flacco et al., 2012).

**Table 2:** Avoidance results of our method and method (Flacco et al., 2012).

	**Avoidance time (s)**	**Avoidance number**	**Increased route (mm)**
Our method	5.1	3	976
Method (Flacco et al., 2012)	12.7	5	963

## Discussions

Considering safe interaction, human and robot working in common workspace is usually forbidden. The robot work cell needs to be rather static, in the current industrial applications without sensor surveillance. If a human enters the robot work cell, ensuring the safety of the human and robot by using additional strategy is very significant. Existing applications of coexistence between robots and humans have been commonly used. In these applications, the robot space is optimized to be less in the condition that physical barriers or human isn't involved. In the complex environment, it is likely that humans are mistaken for an operation objects, such as work pieces. Because of the lack of the intelligence, the human environment will be full of danger. In this paper, a Kinect is employed to detect humans, an expert system is used to analyze humans and artificial potential field method is used to avoid humans. The distance detecting human using Kinect is only from 1.0 to 3.0 m and the detection angle is 57 degrees in the horizontal direction and 43 degrees in the vertical direction, these make the detection range very narrow. Therefore, in actual production applications, we can use a few more Kinects or other solutions to overcome this limitation. After introducing the intelligence, it can be achieved that the system provides the human with protection actively.

In the future research, voice will be introduced into the system so that the robot is able to have communication with the human who enters its workspace. In this case, the robot can notice the behavior intention more clearly. Then the avoidance will be more efficient.

## Conclusions

This paper proposed an active collision avoidance algorithm. Thanks to the simple and efficient Kinect sensor, the system can detect the human who enters the workspace of the robot and take different measures according to the movement of the human. The proposed algorithm uses UKF to estimate the skeleton of the human, and employs an expert system to analyze the human behavior. Moreover, the algorithm also uses artificial potential field method to plan a new path for the robot to avoid the human. Finally, the validity of the proposed algorithm is illustrated by the experiments and the experimental results demonstrate that this algorithm has the practical value such as collaborative assembly of human-robot to safeguard the human who enters the working space of the robot.

## Ethics statement

The study was conducted within the law. All participants provided written informed consent.

## Author contributions

GD, SL, FL, and XH contributed conception and design of the study. GD designed the experimental scheme and performed the experiment. SL performed the statistical analysis. FL wrote the first draft of the manuscript. XH wrote sections of the manuscript. All authors contributed to manuscript revision, read, and approved the submitted version.

### Conflict of interest statement

The authors declare that the research was conducted in the absence of any commercial or financial relationships that could be construed as a potential conflict of interest.
